# Influence of Pretreatment Severity Factor and Hammett Acidity on Softwood Fractionation by an Acidic Protic Ionic Liquid

**DOI:** 10.1021/acssuschemeng.2c06076

**Published:** 2023-01-30

**Authors:** Aida R. Abouelela, Pedro Y. S. Nakasu, Jason P. Hallett

**Affiliations:** Department of Chemical Engineering, Imperial College London, LondonSW7 2AZ, United Kingdom

**Keywords:** biomass waste, delignification, fractionation, severity, lignocellulosic biomass, Hammett acidity

## Abstract

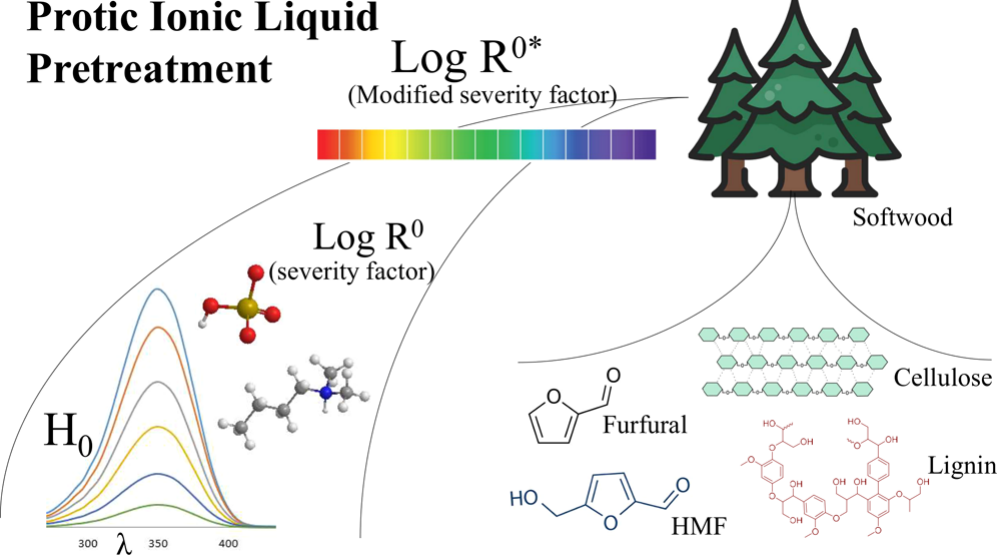

The impact of pretreatment severity in the acidic protic ionic liquid (IL) *N*,*N*-dimethylbutylammonium hydrogen sulfate, [DMBA][HSO_4_] using pine softwood was investigated using a modified severity factor that considers the IL solution acidity based on Hammett acidity. A Box–Behnken experimental design was employed to evaluate pretreatment severity with temperature, pretreatment time, and IL concentration as factors and degree of delignification as the response variable. The optimal pretreatment conditions were found to be at 170 °C, 30 min, and 80 wt % IL, which yielded nearly 90% of delignification and 95% of glucose yield in enzymatic saccharification. The modified severity factor showed an improved correlation with the fractionation indicators relative to the classical pretreatment severity factor, indicating that it can better predict the pretreatment outcomes, particularly for delignification and hemicellulose removal. The fate of hemicellulose, its conversion to humins, and its impact on the precipitated lignin properties were also investigated and correlated to the modified pretreatment severity factor. It was found that such parameters alone cannot be used to predict the fate of dissolved hemicellulose sugars in the IL medium. Furthermore, IL acidity greatly impacts the degradation of the dissolved hemicellulose sugars and the formation of humins.

## Abstract 2

This study entails using protic ionic liquids to process plant biomass waste for further valorization.

## 1 Introduction

Developing an economically viable pretreatment process to obtain a highly digestible cellulose pulp from lignocellulosic biomass is essential for sustainable biofuel production.^1,2^ One of the chemical pretreatment strategies to overcome lignocellulose recalcitrance is the use of ionic liquids (ILs) to deconstruct the lignocellulose polymer matrix made of cellulose (30–50 wt %), hemicellulose (20–40 wt %), and lignin (15–35 wt %).^3^ For the past 20 years, conventional ILs such as 1-ethyl-3-methylimidazolium acetate [Emim][OAc] have shown huge potential for use in a biorefinery scheme, yet questions regarding their cost, solvent recovery, and in situ degradation have been raised as bottleneck challenges.^3,4^ A major advancement in the IL and biomass valorization field is the relatively recent shift toward using protic ILs (PILs), a specific class of ILs that offer several unique advantages compared to their counterparts.^5^ PILs are synthesized via a simple one-step proton transfer reaction between a Brønsted acid (e.g., mineral or organic acids) and Brønsted base (e.g., organic amines), producing a PIL with an available proton for hydrogen bonding.^6^ The simplicity of the reaction and the low cost of the chemical precursors sparked the surge in interest in using PILs as cost-effective solvents, manifesting process commercialization potential.^7,8^ The use of low-cost acidic [HSO_4_]-based PILs to deconstruct a wide variety of virgin and challenging lignocellulosic biomass such as silica-rich biomass and contaminated waste wood was demonstrated in recent studies.^9−11^ The biomass deconstruction mechanism in acidic [HSO_4_]-based PILs depends on deshielding the cellulose polymer via delignification and hemicellulose removal and the subsequent solubilization of lignin and hemicellulose oligomers in the medium.^12^ The delignified cellulose is subsequently hydrolyzed to glucose in a separate enzymatic hydrolysis step. Previous studies have shown a pronounced effect of pretreatment conditions such as temperature, time, cosolvent selection, and water content (severity) on the performance of the pretreatment process using [HSO_4_]-based PILs.^10,13,14^

The use of a pretreatment severity factor (*R*_0_) in IL-based pretreatment methods has been limited compared to its use in aqueous-based pretreatments. The pretreatment severity factor was developed to compare pretreatment yields conducted using different conditions or different aqueous-based pretreatment strategies.^15^ The concept formulation dates back to 1987 as part of a study that evaluates steam-aqueous-based pretreatment methods conducted by Overend and Chornet.^16^ The severity factor relationship trades off temperature against time that results in a similar yield due to the hydrolysis and breakdown of hemicellulose. The kinetics of hemicellulose hydrolysis in an aqueous environment was assumed to follow the first-law concentration dependence and the reaction constant has Arrhenius-type dependence temperature. Therefore, time (*t*) and temperature (*T*) were both combined into a single factor to express the reaction ordinate, the *P*-factor, which was later renamed *R*_0_ using the following equation
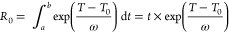
1in which *R*_0_ is the severity factor in minutes, *t* is the time in minutes, *T*_0_ is the reference temperature assigned as 100 °C, *T* is the temperature during pretreatment in °C, and ω is a fitted parameter with a fitted value of 14.75, which is based on the activation energy when pseudo-first-order kinetics are assumed. *R*_0_ has the units of time, however, typically the logarithmic value of *R*_0_ (i.e., log *R*_0_) to easily compare the numerical values of the severity factor. Since acid and base catalysts play a key role in opening the biomass matrix in aqueous-based pretreatment methods, Abatzoglou et al. developed eq 2 to take into account the proton concentration in the pretreatment medium^17^
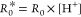
2*R*_0_^*^ is typically referred to as the combined severity factor. The logarithm of the equation yields the following
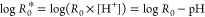
3

The logarithm of the severity factor (log *R*_0_) and the combined severity factor log *R*_0_^*^ have been used as a useful tool to compare and predict the performance of pretreatment strategies in terms of cellulose digestibility with respect to hemicellulose solubilization and lignin removal.^18−20^ Since pH measurements cannot be used for IL solvents due to their nonaqueous nature, expressing the pretreatment severity using log *R*_0_^*^ is not possible. Determining the IL medium acidity requires the use of acidity functions such as Hammett acidity (*H*_0_), which is used to quantify the acidity of nonaqueous systems. To determine IL medium acidity, acidity functions such as Hammett acidity (*H*_0_) are used to quantify the acidity. *H*_0_ is an extension of the pH logarithmic scale to measure Brønsted–Lowry acidity beyond dilute aqueous solutions. The method of determining *H*_0_ consists of measuring the degree of protonation of uncharged indicator bases (In) in a solution in terms of the measurable ratio [In]/[InH^+^].^21^*p*-Nitroaniline has been used as a common base indicator for *H*_0_ determination using UV–vis spectroscopy. *H*_0_ can be expressed as the following
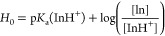
4where p*K*_a_(InH^+^) is the p*K*_a_ value of the protonated *p*-nitroaniline indicator in aqueous solution, and [In] and [InH^+^] are the molar concentrations of the unprotonated and protonated forms of the *p*-nitroaniline indicator in the IL, respectively. Currently, there are only a few literature studies that report *H*_0_ for ILs or PILs, and these studies report *H*_0_ at a specified water content.^22,23^

In addition, to date, only a few studies have evaluated the role of IL or PIL acidity on biomass deconstruction. Cox et al. evaluated the role of IL Hammett acidity in catalyzing the hydrolysis of the β-O-4 ether bond in a lignin model compound using 5 ILs.^24^ The study highlighted that IL acidity (ranging between 1.8 and 2.4) did not correlate with the ability of the IL to catalyze the hydrolysis of the β-O-4 ether bond, indicating an anion interaction effect. Weigand et al. investigated the impact of pretreatment severity in terms of temperature, time, and PIL acidity using triethylammonium hydrogen sulfate [TEA][HSO_4_] and hardwood willow as a feedstock.^25^ The acidity of the PIL was altered using different acid–base ratios during the IL synthesis, and the corresponding pH of a diluted IL solution was used as a high-level indication of the medium’s acidity. Although the use of pH and the acid–base ratio can give an indication of the medium’s acidity, the exact quantification of the IL/H_2_O solution’s acidity was not conducted nor incorporated into the pretreatment severity. Malaret et al. have used the classical pretreatment severity factor to evaluate the deconstruction process of *Eucalyptus*, yet the role of IL concentration and the corresponding acidity was not included in the investigation.^26^

In this study, we aim to evaluate the deconstruction of pine softwood (*Pinus sylvestris*), a viable feedstock for a 2nd generation biorefinery, using *N*,*N-*dimethyl-*n*-butylammonium hydrogen sulfate [DMBA][HSO_4_] aqueous solutions under different pretreatment severity conditions. A Box–Behnken Design (BBD) response surface methodology (RSM) was applied as a tool to design experiments of different severity levels using the three key process variables: temperature, time, and IL concentration. The Hammett acidity for [DMBA][HSO_4_]/H_2_O mixtures used for the pretreatment was measured and *H*_0_ was incorporated into the classical pretreatment severity factor. Although limited to the range of the parameters, this empirical approach better reflects the role of the acidic protic IL and its corresponding solution acidity on the fractionation of the recalcitrant pine softwood feedstock. We presented the correlations between delignification, hemicellulose removal, glucan degradation, and glucose yield via subsequent enzymatic hydrolysis and the modified severity factor with *H*_0_. We also hypothesized about the fate of hemicellulose and lignin during pretreatment and critically assessed the capability of the modified severity factor to predict them.

## 2 Materials and Methods

### 2.1 Materials and IL Synthesis

Chemicals used for IL synthesis, compositional analysis, and enzymatic saccharification were purchased from Sigma Aldrich and used as received with no further purification.

#### 2.1.1 Feedstock

Pine softwood (*Pinus sylvestris*)^27^ was obtained from METLA (Helsinki, Finnish Forest Research Institute). The feedstock was air-dried (7 wt % moisture content), chopped in a wood mill, and sieved to a particle size between 180 and 850 μm (20 + 80 U.S. mesh scale) prior to use, and stored in plastic bags at room temperature.

#### 2.1.2 Synthesis of [DMBA][HSO_4_]

*N*,*N*-Dimethyl-*n*-butylamine (75.9 g, 750 mmol) was cooled in a round-bottom flask within an ice bath; 150 mL of 5 M H_2_SO_4_ (750 mmol) was added dropwise while stirring. The reaction proceeded for at least 5 h with continuous stirring. Excess water was removed using a rotary evaporator. The ionic liquid was recovered as a clear, viscous liquid. The water content of the IL was adjusted to 20 wt % using a volumetric Karl Fischer titrator (V20 Mettler Toledo). ^1^H NMR: δ_H_ (400 MHz, DMSO-*d*_6_)/ppm: 9.24 (s, 1H, N–H), 3.02 (dt, *J* = 12.9, 5.0 Hz, 2H, N–CH_2_), 2.76 (d, *J* = 4.3 Hz, 6H, N–(CH_3_)_2_), 1.64–1.51 (m, 2H, N–CH_2_–CH_2_), 1.30 (h, *J* = 7.4 Hz, 2H, N–CH_2_–CH_2_–CH_2_), 0.90 (t, *J* = 7.4 Hz, 3H, N–CH_2_–CH_2_–CH_2_–CH_3_). ^13^C NMR δ_C_ (101 MHz, DMSO-*d*_6_)/ppm: 55.92 (N–CH_2_), 42.46 (N–CH_3_), 25.82 (N–CH_2_–CH_2_), 19.30 (N–CH_2_–CH_2_–CH_2_), 13.71 (N–CH_2_–CH_2_–CH_2_–CH_3_).

### 2.2 Biomass Pretreatment

Pretreatment, determination of the oven-dried weight, and IL water content measurements were conducted according to the standard operating procedure from our laboratory.^28^ In short, the biomass pretreatment assays were conducted in 10 mL pressure tubes in a convection oven. A certain amount of [DMBA][HSO_4_] of predetermined water content was mixed with 2 g of biomass (on an oven-dried weight basis), corresponding to a biomass-to-solvent ratio of 1:5 g/g. After the oven time elapsed at the specified temperature, the pressure tube was allowed to cool down for at least 20 min. The cellulose-rich pulp was separated from the IL slurry and washed three times using 40 mL of absolute ethanol in a 50 mL Falcon tube. The cellulose pulp was further washed using 24 h Soxhlet extraction with absolute ethanol. The washing protocol with Soxhlet extractors was not conceptualized to reflect the washing process on a commercial plant scale. It was developed aiming to fully recover the IL, while also removing the solubilized lignocellulosic fractions such as lignin and hemicellulose from the cellulosic pulps. Following Soxhlet extraction, the thimbles containing the pulp were emptied into a preweighed falcon tube and washed with 30 mL of water to remove traces of ethanol and ensure that the pulp was kept wet. Approximately 1 g of the wet pulp was taken for moisture content analysis. Cellulose pulp yield was calculated as follows
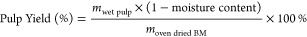
5where *m*_oven dried BM_ is the weight of the oven-dried biomass and moisture content is the water content in the cellulose pulp, determined in the previous section. The ethanol used for the Soxhlet extraction was combined with the ethanol washings from the previous steps and evaporated under vacuum at 40 °C with agitation, leaving a dried IL/lignin mixture (IL black liquor). Different water equivalents were added to the black liquor, corresponding to 30, 20, 15, 10, and 5 mL of distilled water, to precipitate the lignin. The recovered solid lignin was washed three more times with water. The wet lignin was then freeze-dried and the dried lignin was weighed to obtain the lignin yield relative to biomass. Lignin yield relative to the initial lignin content is calculated using the following equation
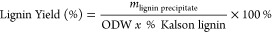
6where *m*_lignin precipitate_ refers to the weight of freeze-dried lignin, ODW refers to the oven-dried weight of the biomass, and Klason lignin refers to the percentage of lignin present in the biomass as determined by compositional analysis.^29^

### 2.3 Hammett Acidity Measurements

IL Hammett acidity was measured using UV–Vis, combining the Beer–Lambert law (eq 6) and a modified form of the Henderson–Hasselbalch equation^30^
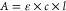
7where *A* is the absorbance, ε is the absorptivity, *c* is the concentration, and *l* is the path length.
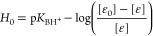
8where *H*_0_ is Hammett acidity, p*K*_BH^+^_ is the basicity constant of the Hammett base, ε_0_ is the absorptivity of the fully unprotonated Hammett base, and ε is the absorptivity of the partially protonated Hammett base.

The p*K*_BH^+^_ value of 4-nitroaniline is 1.00, obtained from the literature.^30^ The Hammett base selected was 4-nitroaniline as it is suitable for the range of Hammett acidities investigated.^30,31^ To determine the extinction coefficient of the fully unprotonated 4-nitroaniline, ε_0_, six concentrations of 4-nitroaniline between 0.1 and 1 mM were prepared in anhydrous DCM. The UV–Vis spectra of these solutions were measured and using eq 7, the extinction coefficient of the 4-nitroaniline, ε_0_ was calculated using the maximum absorbance at 350 nm. The calculated coefficient in this study was 15,572, which is fairly consistent with that reported by Gräsvik et al.^30^ To measure the extinction coefficient (ε) for each [DMBA][HSO_4_]/water mixture, 1 mL of 4-nitroaniline solution was transferred to a 5 mL round-bottom flask. The DCM was removed through rotatory evaporation and 1 mL of the [DMBA][HSO_4_] solution was added. By this method, a series of different 4-nitroaniline concentrations were added to each aqueous IL solution, and a sample for UV–Vis analysis was prepared (a total of 5 4-nitroaniline concentrations per IL solution). The solutions were left overnight to ensure that all of the 4-nitroaniline is dissolved in the IL solution. The UV–Vis spectra of the solutions were later measured and the absorbance of the unprotonated peak at 350 nm was recorded. All Hammett acidity measurements were repeated twice, i.e., by preparing new stock solutions of 4-nitroaniline in DCM and IL, which were then diluted to the desired concentrations. All UV–Vis measurements were performed using a Perkin Elmer Lambda 650, with solutions pipetted into sealed UV-clear quartz cuvettes with a path length of 0.5 cm.

### 2.4 Box–Behnken Design

A 3-level BBD-RSM was conducted using three key pretreatment variables using [HSO_4_]-based protic ILs: pretreatment time, temperature, and IL concentration (Table S1 in the Supporting Information). The corresponding low, central, and midlevels of each variable were: 20, 30, and 40 min (time); 160, 170, and 180 °C (temperature); and 70, 80, and 90 wt % (IL concentration). The response variable was lignin extraction or delignification. A set of 15 trials with 3 replicates at the center point was designed using JMP-Pro. All experiments were carried out in randomized order and each of the trial points was conducted in duplicate. The design of the experiment, data fitting, and analysis of the regression model was conducted using JMP-Pro software. The fit model was evaluated based on the analysis of variance (ANOVA) results of *R*^2^ and *R*^2^ adjusted.

### 2.5 Feedstock and Pulp Characterization

Information about compositional analysis, enzymatic saccharification assays, IL liquor, and lignin characterization can be found in the Supporting Information.

## 3 Results and Discussion

### 3.1 Model Fitting and Impact of Severity Variables on Delignification

The values of the design parameters and the experimental and predicted delignification response for pine softwood are presented in Table 1. The results of the ANOVA (Table S2 in the Supporting Information) analysis showed that the model has a *P* value of <0.0001 and an *F* value of 74. This demonstrates that the model has a 99% level of confidence (α = 0.01) and all effects can be described with a quadratic model. The model fitted the data with an *R*^2^ of 0.986 for the delignification response, suggesting that there is a strong correlation coefficient between the data. The model also exhibits highly adjusted *R*^2^ and predicted *R*^2^ values of 0.973 and 0.965, respectively, suggesting that the model has a high predictive ability and that it is unsuitable for explaining only a 3% variation in response. The highly comparable results in each run between the experimental and predicted delignification values can be seen in Table 1. The statistical significance of the model term coefficients determined by the Student *t*-test and *p*-test is shown in Table 2. The analysis showed that all of the quadratic model terms are statistically significant (*p* < 0.01), and therefore, no term can be eliminated from the model. The second-order polynomial model fit to the experimental data predicts the response using the following equation
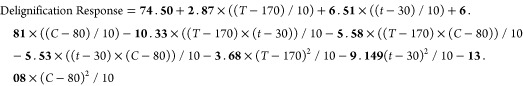
9

**Table 1:** Operating Conditions Determined by the RSM-BBD Method, Composition and Enzymatic Hydrolysis Yield for Biomass Pretreatment Using [DMBA][HSO_4_], and the Corresponding Experimental and Predicted Delignification Responses

	operating conditions			delignification (%)	
run	*T* (°C)	*t* (min)	*C*(wt %)	exp	pred^a^
1	170	20	90	73.74	73.36
2	180	20	80	70.27	67.51
3	180	40	80	62.74	60.20
4	170	30	80	88.68	88.39
5	160	30	70	38.41	36.16
6	160	20	80	40.43	43.17
7	160	40	80	57.98	60.77
8	160	30	90	68.85	67.24
9	170	20	70	34.11	34.27
10	180	30	90	65.01	67.81
11	170	30	80	89.07	88.39
12	170	40	90	59.35	59.19
13	180	30	70	56.28	59.36
14	170	40	70	58.59	58.74
15	170	30	80	88.10	88.39

a Predicted values based on the delignification response quadratic model from eq 9.

**Table 2:** Model Fit Coefficients and the Statistical Significance of the Model Terms of Equation 9

term	coefficient	std error	*t* value	prob > |*t*|
intercept	74.50	0.73	101.44	<0.0001
*T*	2.87	0.45	6.39	<00001
*t*	6.51	0.45	14.48	<0.0001
*C*	6.81	0.45	15.14	<0.0001
*T* × *t*	–10.34	0.64	–16.25	<0.0001
*T* × *C*	–5.59	0.64	–8.78	<0.0001
*t* × *C*	–5.53	0.64	–8.70	<0.0001
*T*^2^	–3.68	0.66	–5.57	<0.0001
*t*^2^	–9.15	0.66	–13.82	<0.0001
*C*^2^	–13.08	0.66	–19.77	<0.0001

**Figure 1:**
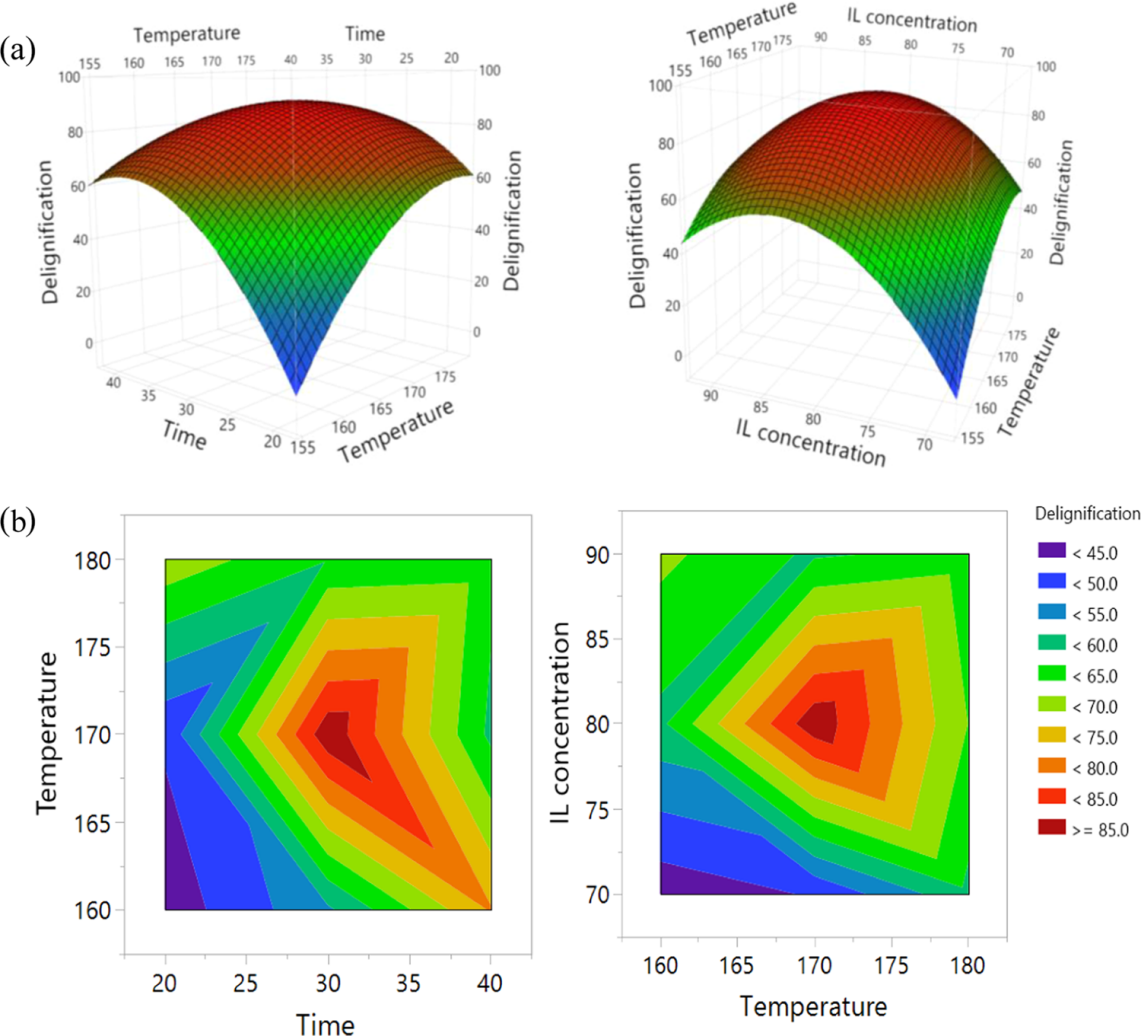
(a) BBD-RSM response surface graphs and the (b) corresponding counter plots at the center point. At the center point, IL concentration = 80 wt % (left) and time = 30 min (right).

The synergetic effect of each variable is illustrated by the 3D response surfaces and the contour plots, as shown in Figure 1a,b. The 3D delignification responses of *T*–*t* and *T*–*C* variables exhibited steep curved surfaces, which highlights the sensitivity of the response in the design space. This is also evidenced by the values in Table 1 where the experimental and predicted delignification efficiency varied from 35 to 88%, depending on the process variable combination (i.e., pretreatment severity). The contour plots highlight the regions where the maximum delignification response (≥70%) is predicted. We can see that the region (in blue) where high delignification is achieved is small, suggesting the sensitivity of the model to the design variables. This is in alignment with the results of the 3D response surface. The results reflect that optimal fractionation in acidic [HSO_4_] PILs can only be achieved in a narrow window, particularly at high temperatures (>120 °C).

In the [HSO_4_]-based protic IL biomass fractionation process, low delignification is typically achieved under two operating conditions: (i) low pretreatment severity where the process variables are not severe enough to effectively extract the native lignin from the biomass or (ii) very high pretreatment severity conditions used where the biomass is “overcooked” allowing the redeposition of extracted lignin fragments and sugar dehydration products onto the cellulose surface in the form of pseudo lignin or humins.^12,32^ The formation of humins in high-severity conditions is typically also associated with hemicellulose sugar dehydration products, which undergo polycondensation reactions forming complex aromatic structures that can redeposit on the cellulose fibers and/or coprecipitate with the lignin fraction.^33^ Optimal delignification is achieved when the process variables are in a delicate balance to achieve the sweet spot where enough native lignin is extracted from the biomass with minimal deposition of humins on the cellulose surface. Runs 6 and 9 demonstrate low-severity conditions where a combination of low temperature, short residence time, or low IL concentration resulted in low delignification values of 40.4 and 34.1%, respectively. The design parameters that provide the optimal delignification of ∼88% for pine softwood were at 170 °C for 30 min and using 80 wt % [DMBA][HSO_4_], which represents the central point in the BBD design (run 4, 11, 15). Other process conditions also achieved high delignification performance (>70%) using 170 °C, 20 min, and 90 wt % [DMBA][HSO_4_] (run 1) and 180 °C, 20 min, and 80 wt % (run 2). Runs 3, 10, and 12 illustrate how high-severity conditions are due to a prolonged residence time, high temperature, or high IL concentration. Other conditions resulted in midrange delignification values from 59 to 62%.

### 3.2 Modified Pretreatment Severity Factor and Its Impact on Fractionation

**Figure 2:**
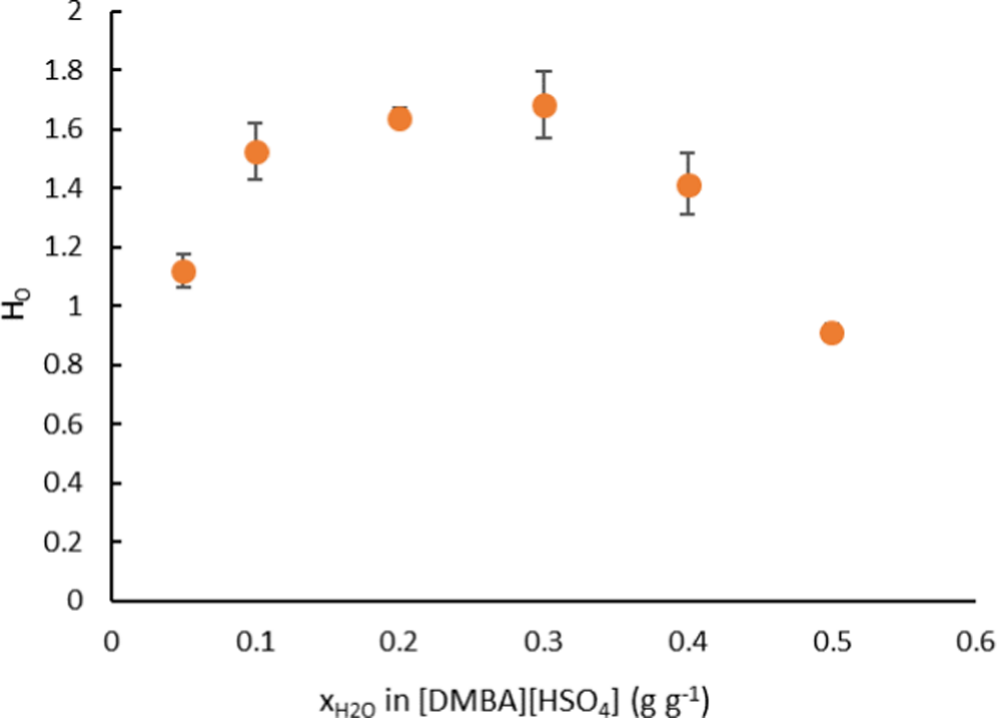
Hammett acidity value for [DMBA][HSO_4_]/water mixtures.

In acidic protic IL systems such as [DMBA][HSO_4_], the acidity of the medium, and therefore the severity of pretreatment, can be controlled by adjusting the acid–base ratio during the synthesis or by changing the IL water content.^25^ As part of the BBD experimental design, the IL water content was considered as a factor and it was therefore varied to scrutinize the impact of water concentration on the acidity behavior of the pretreatment medium. Hammett acidity is a function that is typically used to measure the acidity of nonaqueous media.^34^ It must be noted that a lower *H*_0_ value means the solution is more acidic due to the higher tendency of the solution to donate protons, and thus a “higher” solution acidity (*H*_0_ is analogous to pH, as the two scales converge in aqueous solutions). The *H*_0_ values of [DMBA][HSO_4_]/water mixtures (*x*_H_2_O_) = 5–50 wt % are presented in Figure 2. [DMBA][HSO_4_]/water mixtures had the lowest acidity with 20–30 wt % water, with *H*_0_ values of 1.64 and 1.68, respectively. Increasing water concentrations above 30 wt % showed a dramatic decrease in *H*_0_, indicating that excess amount of water increased the proton transfer ability of the medium.

At 5 wt % water in [DMBA][HSO_4_], the medium acidity increased noticeably. The effect is attributed to the lower solvation of the IL ions at a low water content (solvation limit around *x*_H_2_O_ = 20 wt %, corresponding to 75 mol %),^35^ which increases the proton chemical activity of sulfuric acid nonlinearly due to a significantly reduced proton solvation energy in the absence of more basic water molecules.^35^ It is interesting to note that the acidity trend of [DMBA][HSO_4_] water mixtures matches the reported results by De Gregorio et al. using 1-butyl-3-methylimidazolium hydrogen sulfate [C_4_C_1_im][HSO_4_], an aprotic IL, when 10 and 20 mol % excess amounts of acid were used during the synthesis step (i.e., acid–base ratios of 1.1 and 1.2).^36^

In this study, the IL concentrations used were 90, 80, and 70 wt %, which correspond to solution acidities of *H*_0_ = 1.50, 1.64, and 1.68, respectively. We incorporated the Hammett acidity function of the IL solutions (eq 6) into the classical pretreatment severity factor logarithmic expression (log *R*_0_). The resulting expression is referred to as the modified pretreatment severity factor in its logarithmic format (log *R*_0_^*^) calculated as in eq 10
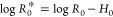
10

It should be emphasized that classical and modified severity factors are *R*_0_ and *R*_0_^*^; however, their logarithm values will be used throughout the study to easily compare the resultant numerical numbers. The modified pretreatment severity factor log *R*_0_^*^, the severity factor log *R*_0_, cellulose pulp yield, structural composition of the recovered cellulose pulps (normalized), experimental delignification values obtained by compositional analysis, as well as the enzymatic hydrolysis of the cellulose pulps to glucose are presented in Table 3. The BBD-RSM experimental runs presented in Table 1 are renumbered in Table 3 based on the modified pretreatment severity factor to facilitate the comparison and subsequent interpretation.

**Table 3:** Cellulose Pulp Structural Composition after Pretreatment with [DMBA][HSO_4_] and the Enzymatic Hydrolysis Glucose Yield

#	*T* (°C)	*t* (min)	*C*(wt %)	log *R*_0_^*^	log *R*_0_	cellulose pulp yield (wt %)	glucan	hemicellulose	lignin^a^	delignification (%)	glucose yield (%)
1	180	40	80	2.32	3.96	27.1	16.8	0	10.3	62.7	64.5
2	180	30	90	2.31	3.83	34.8	23.1	0	11.7	65.01	61.8
3	180	30	70	2.15	3.83	39.1	24.2	0	14.9	56.3	77.7
4	170	40	90	2.14	3.66	37.8	25.6	0	12.2	59.4	66.7
5	180	20	80	2.02	3.66	42	34.3	0	7.8	70.3	55.2
6	170	40	70	1.98	3.66	43.8	30.1	1.6	12.1	58.6	68.6
7	170	30	80	1.90	3.54	43.4	39.1	0.7	3.6	88.10	94.9
8	170	30	80	1.90	3.54	43.4	39.8	0.6	3	88.7	95.1
9	170	30	80	1.90	3.54	43.9	39.7	0.7	3.2	89.1	94.1
10	170	20	90	1.84	3.36	50.5	37.6	0	12.9	73.7	90.1
11	160	40	80	1.73	3.37	44.6	34.2	1.3	9.1	57.9	80.5
12	160	30	90	1.72	3.24	47.5	39.1	0.8	7.5	68.8	87.6
13	170	20	70	1.68	3.36	60.7	38.2	2.9	19.7	34.1	32.7
14	160	30	70	1.56	3.24	57.1	36.8	3.6	16.7	38.4	44.4
15	160	20	80	1.43	3.07	64.6	42.2	4	18.4	40.4	23.1
UB^b^							44.5	25	29.9		1.97

a Sum of acid-insoluble lignin (AIL) and acid-soluble lignin (ASL) obtained from compositional analysis.

b Untreated pine softwood.

On the basis of the different combinations of process variables used in the BBD-RSM experiments, the modified pretreatment severity factor log *R*_0_^*^ ranged from 1.4 to 2.3, representing the lowest and highest severity conditions, respectively. On the other hand, the pretreatment severity factor spanned from log *R*_0_ 3.1–4. The limitations of the use of the severity factor, log *R*_0_, without the incorporation of the IL, acidity can be visualized in Table 3. Pairwise inspection of runs 12 and 14; 10 and 13; and 4 and 6; which had identical severity factors of 3.24, 3.36, and 3.66, respectively, showed that the runs have resulted in vastly different delignification responses due to the different medium acidity (i.e., wt % IL concentration used). For instance, runs 10 and 13 both had an identical log *R*_0_ of 3.36, whereas the corresponding delignification varied significantly from 73 to 34%, respectively. The use of log *R*_0_^*^—which considers the IL acidity— showed different values for runs 10 and 13 to 1.84 and 1.68, which better reflect the large difference in delignification performance.

**Figure 3:**
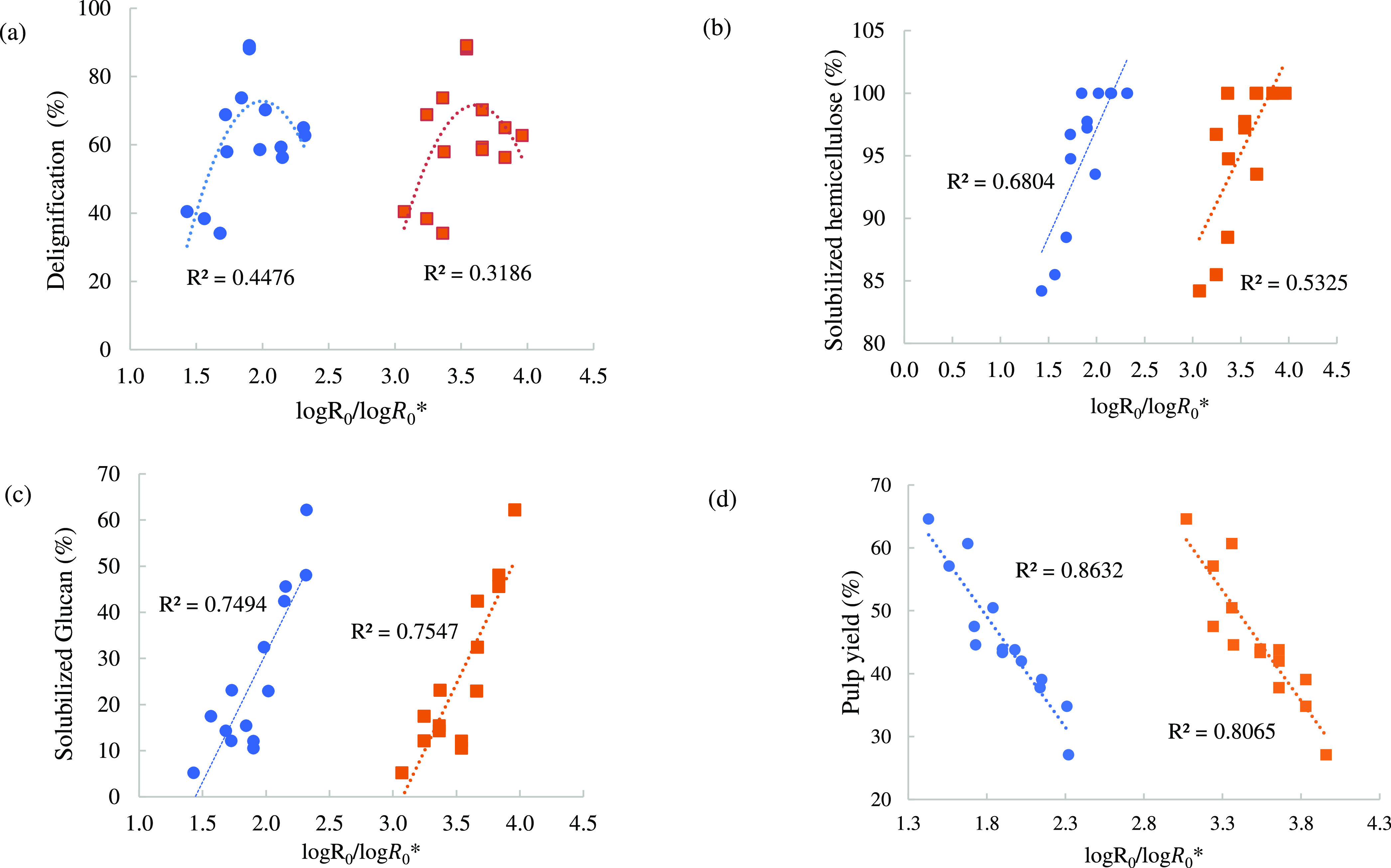
Pretreatment severity factor correlation with (a) lignin removal, (b) solubilized hemicellulose, (c) solubilized glucan, and (d) pulp yield using log *R*_0_ and log *R*_0_^*^.

The fit of the classical severity factor log *R*_0_ and the modified severity factor log *R*_0_^*^ with delignification, hemicellulose and glucan solubilization, and the pulp yield obtained from the compositional analysis data is presented in Figure 3. The correlations of the two severity factors with delignification were expressed as quadratic fits considering the results obtained by the BBD-RSM analysis where delignification showed a steep curvature. On the other hand, the severity factors correlated linearly with polysaccharide solubilization and the pulp yield. Comparing the fittings between log *R*_0_^*^ and log *R*_0_, we can see that fitting the data against log *R*_0_^*^ improved the fit for hemicellulose solubilization, delignification, and pulp yield, whereas the fit for glucan solubilization seemed to be largely unchanged between the classical and the modified severity factors. The more significant improvement in hemicellulose removal and delignification fittings upon introducing *H*_0_ reflects the higher sensitivity of hemicellulose and lignin removal by the acidity of the medium. Kim et al. (2014) improved the correlation between the severity factor and pretreatment parameters (e.g., xylan solubilization) by showing that temperature plays a bigger role in pretreatment efficiency than the commonly used severity factor.^37^ Wyman and Yang also verified the improved fitting from the classic (log *R*_0_) and combined severity factor (log CS) on xylan solubilization upon dilute acid and hydrothermal pretreatment.^38^

Glucan solubilization (Figure 3c) ranged between 5 and 20% when the pretreatment severity was <1.9. At severity levels > 1.9, we notice a significant increase in glucan degradation reaching up to 60% losses at the highest severity of 2.32. Glucan losses up to 10% are normal as some glucan is considered part of the hemicellulose polymers.^39^ However, a higher degree of glucan degradation indicates that cellulose is being hydrolyzed due to the high-severity conditions. The optimal conditions for lignin and hemicellulose removal and minimal glucan degradation correspond to 170 °C, 30 min, and 80 wt % IL (log *R*_0_^*^ of 1.9), which delignified 88%, removed 97% of hemicellulose, and solubilized only 10% of cellulose in the biomass.

There was a negative correlation between the cellulose pulp yield (i.e., recovered cellulose-rich residues after pretreatment) and both log *R*_0_ (*R*^2^ = 0.806) and log *R*_0_^*^ (*R*^2^ = 0.863) (Figure 3d). The correlation is associated with the solubilization of all of the biomass components, lignin, hemicellulose, and cellulose at higher severity values which, conversely, yields a low amount of pulp. The low cellulose pulp yields despite the relatively high delignification reflect the severe degradation of cellulose along with the extraction of hemicellulose and lignin. It should be noted that the remaining ∼30% lignin in the cellulose pulp at such high-severity conditions reflects both the nonextracted native lignin and the redeposited humins. From the residual lignin content in the recovered cellulose pulps, we can see that only a few operating conditions effectively extracted lignin, achieving ≥ 70% delignification, i.e., runs 5, 10, and 12 the central BBD runs 7–9. High-severity experiments with log *R*_0_^*^ 2.14–2.32 resulted in a milder delignification of 55–65%.

**Figure 4:**
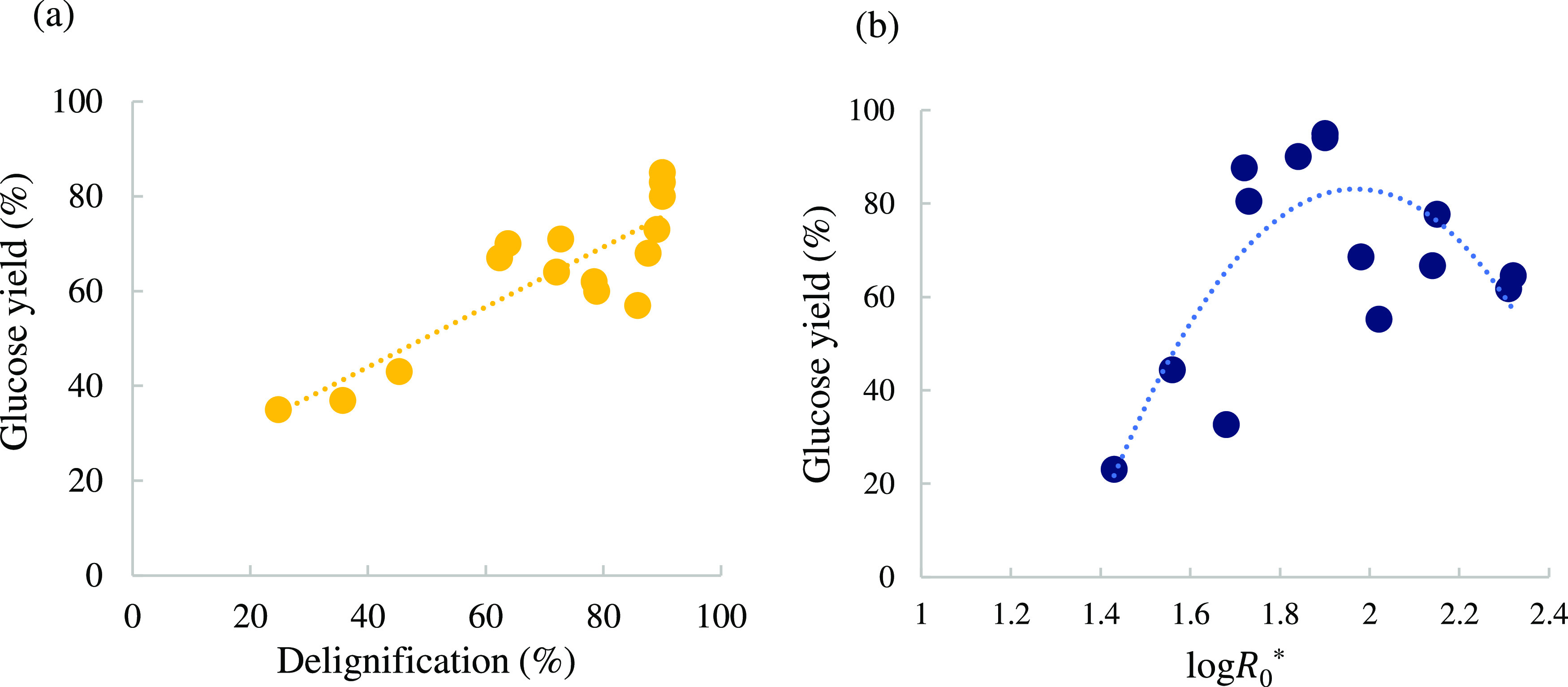
Correlation between glucose yield and delignification (a) and the modified severity factor (b). Enzymatic hydrolysis was conducted for 72 h and pretreatment conditions are summarized in Table 3.

Glucose yields from enzymatic hydrolysis of recovered cellulose pulps show a high variation in response ranging between 23 and 95% relative to the theoretical maximum (Figure 4a). There is an overall positive correlation between glucose yield and lignin extraction, which was also observed in previous studies.^10,40^ The positive correlation can be related to the higher surface area, therefore exposure, of cellulose substrate, allowing easier access of enzymes. The variation in glucose yield depending on the modified severity factor is presented in Figure 4b. Once delignification—which presents a quadratic correlation with log *R*_0_^*^ (Figure 3a)—and glucose yield present a linear relationship (Figure 4a), it is reasonable to think of a nonlinear relationship between the glucose yield and the modified severity factor. Similar to the delignification trend, glucose release from cellulose pulp was the highest at log *R*_0_^*^ 1.7–1.9 ranging between 80 and 90% relative to the theoretical maximum. At a high severity factor, the glucose yield drops to <70% whereas at a low severity factor, glucose yields are <40%.

There are experimental conditions where delignification values alone do not entirely reflect and explain the glucose yields obtained. For instance, runs 14 and 15 resulted in very similar delignification values of 38 and 40%, respectively, whereas the glucose yields corresponded to 44 and 23%, respectively. Similarly, the central point condition (170 °C, 30 min, 80 wt % IL) resulted in a near-quantitative glucose yield of ∼95% (log *R*_0_^*^ of 1.9, delignification 88%) and run 10 (170 °C, 20, and 90 wt % IL) also resulted in a 90% glucose yield, despite the slightly lower delignification achieved (i.e., 73%). Such variation highlights the importance that other factors could have on glucose yield such as the chemical and physicochemical features of the residual lignin on the cellulose pulp (impacted by the pretreatment severity). A similar effect was also observed using softwood and waste wood feedstocks where it was reported that the properties of residual lignin on the cellulose pulp (e.g., extent of condensation) had a strong impact on the extent of glucose release from cellulose.^10,41^

### 3.3 Fate of Hemicellulose and Lignin during Pretreatment

The composition of the soluble sugar monomers, furfural, levulinic acid, and 5-HMF found in the IL liquor relative to the biomass (mg g^–1^) is presented in Table 4. Optimal delignification conditions (i.e., central point run 7–9) have yielded 2.5 wt % furfural and 3.2 wt % 5-HMF relative to untreated biomass. Although these yields are low, they are still promising especially as the process is not optimized to produce these compounds. Current industrial furfural production processes depend on the use of agricultural residues, such as corncobs in China and bagasse in South Africa.^42^ Quaker Oats Technology in China uses continuous fixed bed reactors followed by azeotropic distillation to isolate furfural.^42^ The yield of furfural in the process is 4–12% with respect to the dry-weight biomass. Other more advanced processes such as SupraYield yield 50–70% furfural; however, the process uses higher operating temperatures (240 °C) which allows more efficient conversion of sugars to furfural.^42^ Therefore, with slight modification and optimization of operating conditions, the ionoSolv process offers good potential to be used for furfural production. 5-HMF is also a key platform chemical in the biorefinery; however, the production of 5-HMF in high yield as well as its separation from the reaction medium is far more challenging than furfural, and therefore industrial production of 5-HMF remains at an early stage of development.^43−45^

**Table 4:** IL Liquor Composition in Terms of Monomeric Sugars and Dehydration Products

						concentration (mg g^–1^ of biomass)					
#	*T* (°C)	*t* (min)	*C*(wt %)	log *R*_0_	log *R*_0_^*^	glucose	xylose	mannose	furfural	HMF	levulinic acid
1	180	40	80	3.96	2.32	50.2	70.2	4.7	16.0	23.9	41.9
2	180	30	90	3.83	2.31	33.1	3.0	1.5	6.7	33.1	32.5
3	180	30	70	3.83	2.15	60.2	15.4	8.9	9.5	20.0	24.8
4	170	40	90	3.66	2.14	32.3	14.9	8.6	22.4	28.6	4.3
5	180	20	80	3.66	2.02	38.7	49.8	29.2	17.7	21.2	13.1
6	170	40	70	3.66	1.98	49.2	29.7	17.3	15.2	23.0	9.3
7	170	30	80	3.54	1.90	48.2	66.8	39.1	26.6	31.6	10.4
8	170	30	80	3.54	1.90	48.8	67.9	39.5	25.7	33.4	18.8
9	170	30	80	3.54	1.90	48.6	67.5	39.8	26.0	32.5	13.2
10	170	20	90	3.36	1.84	24.9	46.0	27.0	16.1	17.9	7.9
11	160	40	80	3.37	1.73	24.7	31.0	18.2	13.4	14.6	10.1
12	160	30	90	3.24	1.72	19.0	32.1	16.4	15.2	16.4	8.0
13	170	20	70	3.36	1.68	26.4	87.0	51.1	13.8	16.1	20.6
14	160	30	70	3.24	1.56	29.3	74.0	43.5	11.5	11.8	6.5
15	160	20	80	3.07	1.43	16.8	66.9	39.3	10.1	6.3	4.3

Furfural and 5-HMF are considered fermentation inhibitors in aqueous-based pretreatment processes such as steam explosion and dilute acid pretreatment as cellulose is not isolated/filtered prior to the enzymatic hydrolysis or fermentation step. Fractionation methods such as [HSO_4_]-based PIL offer a key advantage in this regard, where fermentation inhibitors (i.e., sugar dehydration products) are formed during the pretreatment; however, they do not enter the downstream steps.^5^

The unaccounted fraction is calculated based on the balance difference between the sum of hemicellulose residual in the cellulose pulp and detected hemicellulose compounds in the liquor. Brandt-Talbot et al. also reported high unaccounted hemicellulose of 60% while performing mass balances at high-severity conditions (i.e., 120 °C, 20 wt %, [TEA][HSO_4_] for 24 h, *Miscanthus* feedstock)%.^12^ A mass balance is presented in Table 5, and it shows the monomeric sugars (glucose, xylose, mannose), C5 and C6 dehydration products (furfural, 5-HMF), and hydrolysis products (levulinic acid and formic acid) conducted relative to the initial hemicellulose content in the untreated softwood pine. That is, furfural calculation (in mol %) was based on the pentosan content in the raw pine. Likewise, 5-HMF and levulinic acid yields (in mol %) were calculated based on the original glucan content in the raw biomass. Pretreatment using [DMBA][HSO_4_] removed > 80 mol % of hemicellulose in all investigated conditions, and the detection of hemicellulose sugars in the IL liquor after treatment did not exceed 18%. Across all operating conditions, the unaccounted hemicellulose corresponded to the most significant part of the mass balance, ranging between 60 and 86%, even when considering the dehydration product yields.

**Table 5:** Hemicellulose Balance in IL Liquor in the Form of Dissolved Sugar Monomers and Dehydration Products, Residual Hemicellulose in the Recovered Cellulose Pulp, and the Remainder Unaccounted

		conditions						e
run	log *R*_0_^*^	*T* (°C)	*t* (min)	*C*^a^(wt %)	dissolved hemicellulose sugars^b^ (%)	dissolved dehydration products^c^ (%)	hemicellulose in pulp^d^ (%)	unac counted^e^ (%)
1	2.32	180	40	80	13.00	14.38	1.40	71.22
2	2.31	180	30	90	0.78	12.21	0.00	87.01
3	2.15	180	30	70	4.22	9.65	0.00	86.13
4	2.14	170	40	90	4.08	12.74	4.50	78.68
5	2.02	180	20	80	13.72	10.93	0.00	75.35
6	1.98	170	40	70	8.16	10.09	1.00	80.75
7	1.90	170	30	80	18.39	15.29	0.80	65.52
8	1.90	170	30	80	18.65	20.10	1.10	60.15
9	1.90	170	30	80	18.55	11.15	1.10	69.20
10	1.84	170	20	90	12.67	9.22	0.00	78.11
11	1.73	160	40	80	8.55	8.02	0.00	83.43
12	1.72	160	30	90	8.43	8.67	5.50	77.40
13	1.68	170	20	70	23.98	9.68	2.70	63.64
14	1.56	160	30	70	20.41	6.50	2.30	70.79
15	1.43	160	20	80	18.43	4.74	6.60	70.23

a *H*_0_ values for 90, 80, and 70 wt % aqueous [DMBA][HSO_4_] solution is 1.50, 1.64, and 1.68, respectively.

b Sum of xylose and mannose sugars in IL liquor.

c Sum of furfural, 5-HMF, and levulinic acid in IL liquor; formic acid was detected below the quantification limit.

d Sum of hemicellulose components in the pulp.

e Remainder balance.

The more plausible route for the fate of solubilized hemicellulose sugars in protic [HSO_4_]-based ILs is their transformation into soluble and insoluble humins/pseudo lignin.^48^ The soluble structures remain in the IL liquor during pretreatment, whereas the insoluble part redeposits onto the cellulose surface. A hypothesized reaction pathway starts from the dehydration of C5 and C6 sugars to furfural and 5-HMF.^49^ Both furfural and 5-HMF—and their dehydration products such as formic and levulinic acids—can then be converted to other aromatic compounds, which are key precursors for humin formation via polymerization reactions. The largest component of hemicellulose polymer in pine softwood used in this study is mannan (a C6-based carbohydrate) with a content of 13.5% (55% of total hemicellulose polymer), followed by xylan (C5) with 5.1% (i.e., 20% of total hemicellulose), while arabinan (C5) and galactan (C6) account equally for the remaining balance.

According to the mass balance, the two conditions with the highest modified severity factors are: log *R*_0_^*^ 2.32 (180 °C, 30 min, 80 wt % IL) and log *R*_0_^*^ 2.31 (180 °C, 30 min, 90 wt % IL). These resulted in “unaccounted” hemicellulose fractions of 71 and 87 mol %, respectively, with the main variation stemming from the solubilized sugars in the IL medium. The discrepancy is also clear when comparing the detected soluble sugars in the IL medium between the two runs. A study by Xu et al.^50^ with the aprotic IL [BMIM]Cl on the Cr(III)-catalyzed production of 5-HMF has shown that hexose sugars can dehydrate to produce 5-HMF, which, in turn, can be converted into soluble and insoluble humins. In the absence of water, more insoluble humins were formed, and with increasing water content, the soluble humin content was higher. The same rationale can be applied to the ionic liquid system employed in this work, where the acidic [HSO_4_]^−^ anion provides hydronium ions that catalyze the dehydration of C5 and C6 sugars from hemicellulose. The insoluble humins were likely accounted as Klason lignin; however, the soluble humins remained unaccounted for.

At lower or higher severity conditions than 1.9, there were fewer dehydration products found in the IL liquor, which is either due to lower initial extraction of hemicellulose (low-severity conditions) or polycondensation reactions of these products (high-severity conditions). For example, 20% of hemicellulose was quantified as sugar monomers (xylose and mannose) in runs 13 and 14 (log *R*_0_^*^ of 1.68, 1.56); however, only 6–10% dehydration products were formed as the formation of furfural, 5-HMF, and levulinic acid typically requires high-severity conditions.^51^ On the other hand, under high-severity conditions where log *R*_0_^*^ > 1.9, the dehydration products yield varied from 6 to 11%. Run 4 (log *R*_0_^*^ of 2.14) has the 2nd highest dehydration product yield after the central point; however, there were only 4% detectable monosaccharide sugars in the IL liquor. This indicates the excess of sugars formed during dehydration, which subsequently were polymerized and condensed, forming humins.

Sipponen and co-workers studied the impact of hot water pretreatment severity on the generation of humins from wheat straw, and their study elucidated that higher severity induced the accumulation of more humins within the temperature range of 170–200 °C.^52^ Guo et al. also reported that high-severity conditions promoted the formation of aromatic-rich humin structures from the aliphatic structures at lower severity.^53^

**Figure 5:**
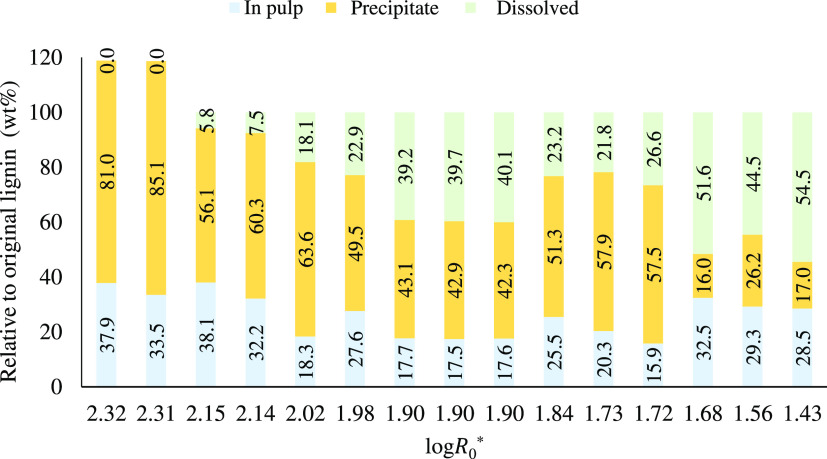
Lignin mass balance. Pine softwood was pretreated according to the conditions presented in Table 4. Values were calculated relative to the initial lignin content in the untreated biomass. Pulp lignin is the sum of acid-insoluble lignin and acid-soluble lignin obtained from the compositional analysis. Precipitated lignin is the lignin obtained after water addition as an antisolvent. Dissolved lignin is the lignin remaining in the IL liquor calculated as the remaining balance.

Lignin mass balance as a function of the modified severity factor is shown in Figure 5 for pine softwood was pretreated according to the conditions presented in Table 4.

The two runs that corresponded to the highest severity (log *R*_0_^*^ 2.32 and 2.31) have exceptionally high lignin yields of >80% resulting in a mass balance that exceeds 100% indicating the formation of humins. On the other hand, the lignin yield obtained during optimal delignification conditions (log *R*_0_^*^ 1.9) was 42%.

The molecular weight characterization of the precipitated lignin is presented in Table 6. Runs 1 and 2 have high polydisperse lignins with PDI values of 8.5 and 8.3, respectively. The high PDI values indicate the highly diverse and branched structure of the lignin and humins formed via polycondensation reactions of sugar intermediates, causing broad molecular weight distributions. Interestingly, the average *M*_w_ of the lignin produced in runs 1 and 2 was 62,000 g mol^–1^, which is lower than the average *M*_w_ of the lignin extracted in the optimal delignification conditions of 69,000 g mol^–1^. However, the PDI of the precipitated lignin at the optimal condition was ∼5, indicating that lignin is less modified compared to the lignin/humins precipitated at high-severity conditions.

**Table 6:** *M*_w_, *M*_n_, and PDI for Extracted Precipitated Lignin at Different Severity Conditions

run	*T* (°C)	*t* (min)	IL (wt %)	log *R*_0_^*^	*M*_n_ (Da)	*M*_w_ (Da)	PDI (*M*_w_/*M*_n_)
1	180	40	80	2.32	754	6256	8.3
2	180	30	90	2.31	736	6229	8.5
3	180	30	70	2.15	685	2904	4.2
4	170	40	90	2.14	1457	6864	4.7
5	180	20	80	2.02	706	3960	5.6
6	170	40	70	1.98	1356	5490	4.0
7	170	30	80	1.90	1435	6803	4.7
8	170	30	80	1.90	1337	6832	5.1
9	170	30	80	1.90	1453	7266	5.0
10	170	20	90	1.84	1332	4019	3.0
11	160	40	80	1.73	882	5695	6.5
12	160	30	90	1.72	732	4506	6.2
13	170	20	70	1.68	1272	3372	2.7
14	160	30	70	1.56	705	2623	3.7
15	160	20	80	1.43	838	3143	3.8

On the other hand, low-severity conditions such as runs 11 and 13 showed low PDI values of 3 and 2.7, respectively. It is interesting to note that when the pretreatment was conducted at 180 or 160 °C, the number average molecular weight *M*_n_ was <1000 for all runs. At 180 °C, the lignin chains were short, yet they had high *M*_w_ due to the high-severity conditions (log *R*_0_^*^ > 2) and the competing polycondensation and fragmentation reactions of the extracted lignin/humins. On the other hand, at 160 °C fractionation runs (log *R*_0_^*^ values from 1.4 to 1.7), the lignin chains also had low *M*_n_, which reflects the extraction of smaller polymer chains due to the low delignification, or similarly, competing polycondensation and fragmentation reactions.

## 4 Conclusions

The modified severity factor has shown an improved correlation with the fractionation indicators (degree of delignification, pulp yield, and hemicellulose removal) relative to the classical pretreatment severity factor. Therefore, it increases the level of precision and significance of the prediction of pretreatment outcomes. The optimal pretreatment conditions—which happened to be in the center point of the experimental design, increasing its credibility—allowed a fast pretreatment (30 min) that produced a highly digestible (>90% glucose yield) cellulose-rich pulp. The fate of hemicellulose and lignin was also investigated. Hemicellulose conversion to humins was found to impact the precipitated lignin properties, such conversion could also be correlated to the modified pretreatment severity factor. We concluded that the modified severity factor alone cannot be used to predict the fate of solubilized hemicellulose sugars in the IL medium and that IL acidity has a high impact on the degradation of the dissolved hemicellulose sugars and the formation of humins. Suppressing these hemicellulose side reactions is key to improving the ionoSolv pulp quality.

## Supporting Information Available

Detailed procedure about compositional analysis, enzymatic saccharification assays, and IL liquor and lignin characterization; experimental factors, levels, and the code of variables chosen for Box–Behnken design; and the ANOVA results for the model fit (PDF)

## Abbreviations

ANOVA: analysis of variance
BBD: Box–Behnken design
C5: pentose
C6: hexose
[DMBA][HSO_4_]: *N*,*N*-dimethylbutylammonium hydrogen sulfate
DMSO: dimethyl sulfoxide
[Emim][OAc]: 1-ethyl-3-methylimidazolium acetate
HPLC: high-performance liquid chromatography
5-HMF: 5-hydroxymethyl furfural
IL: ionic liquid
NREL: National Renewable Energy Laboratory
PDI: polydispersity index
PIL: protic ionic liquid
RSM: response surface methodology
[TEA][HSO_4_]: triethylammonium hydrogen sulfate
