# Supplementary material for: Influence of Pretreatment Severity Factor and Hammett Acidity on Softwood Fractionation by an Acidic Protic Ionic Liquid
Source: ACS Sustain Chem Eng. 2023 Jan 30;11(6):2404–15. doi: 10.1021/acssuschemeng.2c06076 (PMC9930189; doi:10.1021/acssuschemeng.2c06076)
Supplement: Supplementary file 1 — sc2c06076_si_001.pdf [file sc2c06076_si_001.pdf]

Electronic Supplementary Information

The influence of Pretreatment Severity Factor and Hammett Acidity on Softwood Fractionation by an Acidic Protic Ionic Liquid

Aida R. Abouelela[†], Pedro Y.S. Nakasu[†], Jason P. Hallett[†*]

[†]Department of Chemical Engineering, Imperial College London, London SW7 2AZ, United Kingdom

*Corresponding author e-mail: j.hallett@imperial.ac.uk

Number of Pages: 5

Number of Figures: 0

Number of Tables: 2

Compositional Analysis

Compositional analysis was carried out according to a published procedure by the National Renewable Energy Laboratory (NREL) "Determination of Structural Carbohydrates and Lignin in Biomass".[1] Details of the procedure is found in the Supporting Information. Glucan recovery, hemicellulose extraction and lignin extraction were calculated based on the compositional analysis data and all the calculations were done based on weight and the weight percentage of the corresponding biomass component in the untreated biomass or recovered cellulose-rich pulp. Glucan recovery was calculated according to Equation S1:

$$Glucan\ recovery = \frac{Glucan_{pulp} \times Yield_{pulp}}{Glucan_{untreated}} \qquad (S1)$$

Where $Glucan_{pulp}$ is the glucan content in the cellulose pulp, $Yield_{pulp}$ is the over-dried yield of the pulp after ionoSolv processing relative to untreated biomass and $Glucan_{untreated}$ is the glucan content in the untreated biomass.

Hemicellulose extraction was calculated according to Equation S2:

$$Hemicellulose\ extraction = \frac{Hem_{untreated} - Hem_{pulp} \times Yield_{pulp}}{Hem_{untreated}} \quad \text{(S2)}$$

Where $Hem_{untreated}$ is the hemicellulose content of untreated biomass calculated as the sum of all sugars (except glucan) and Hem_{pulp} is the hemicellulose content remaining in the cellulose pulp.

Lignin extraction (i.e. delignification) relative to untreated biomass was calculated following Equation S3:

$$delignification = \frac{Lignin_{untreated} - (Lignin_{pulp} \times Yield_{pulp})}{Lignin_{untreated}} \quad \text{(S3)}$$

Where $\mathrm{Lignin}_{untreated}$ is the lignin content in the untreated biomass and Lignin_{pulp} is the residual lignin content in the recovered cellulose pulp after pretreatment.

Enzymatic Saccharification assay

Enzymatic saccharification assays of cellulose pulps and air-dried untreated biomass were carried out in triplicate, with blanks, according to the NREL protocol "Low solids Enzymatic saccharification of lignocellulosic biomass".[2] The saccharification of the cellulose pulps was performed on their wet state to eliminate the hornification effect[3]. The determination of the moisture content of wet pulps was conducted based on the NREL protocol [4] and ranged from 89 to 90 wt%. All reagents and enzymes were purchased from Sigma Aldrich and used as received. The enzymatic hydrolysis was run for 7 days in an incubator at 50 °C. The glucose yield relative to the theoretical maximum was calculated according to Equation S4:

$$\textbf{Glucose yield}\ (\%) = \frac{(C_{sample} - C_{blank}) \times V_{sample} \times corr_{anhydro} \times Pulp\,Yield}{m_{sample} \times (1 - mc_{sample}) \times Glucan_{untreeated}} \times 100 \qquad \text{(S4)}$$

Where c_{sample} is the glucose concentration of the hydrolysis sample as determined by HPLC, c_{blank} is the glucose concentration of the blank control as determined by HPLC, V_{sample} is the volume of the hydrolysis sample (10 mL), $corr_{anhydrous}$ is a correction factor accounting for the increase in mass of sugars during hydrolysis (0.9 for glucose), m_{sample} is the mass of the hydrolysed sample, mc_{sample} is the moisture content of the hydrolysed sample, and $Glucan_{untreated}$ is the glucan content of the untreated biomass.

Lignin characterization

GPC measurements were performed using an Agilent 1260 Infinity instrument equipped with a Viscotek column set (AGuard, A6000 M and A3000 M). The Agilent 1260 Infinity RID detector was used for detection. GPC grade DMSO containing LiBr (1 $g{\cdot}L^{-1}$) was used as eluent at a flow rate of 0.4 $mL{\cdot}min^{-1}$ at 60 °C. Samples were prepared by dissolving 20 mg lignin in 1 mL eluent and filtering through a 0.2 µm Syringe filter. Ten pullulan standards (Agilent calibration kit, 180 < Mp < 780 000) were used to calibrate the instrument.

IL liquor characterization

IL solutions were analysed by removing 200 mg of the solution with a pipette into an Eppendorf micro centrifuge tube. The exact weight was recorded, ca. 600 mg of water were added, and the exact weight recorded again. The tube was shaken and centrifuged with a VWR MICRO STAR 17R centrifuge at 4 °C and 13.3 G for 10 min to remove any water- insoluble material. The supernatant was pipetted into a HPLC vial and submitted for analysis on a Shimadzu HPLC system with RI and UV/Vis detector and an Aminex HPX-87H column (BioRad, 300 × 7.8 mm) with 0.01 M H_2SO_4 as mobile phase (0.6 $mL{\cdot}min^{-1}$). The column temperature was 55 °C

and acquisition was run for 55 min. Calibration was carried out using glucose, xylose, galactose, arabinose, furfural and 5-HMF standards. Analyte concentrations in the HPLC sample were calculated using the resulting calibration curves. The mass fraction w/w of analytes detected in the IL solution (in mg·g^{-1} of dried biomass) was determined using Equation S5:

$$\text{Solution in IL (w/w)} = \frac{c_{HPLC} \cdot (m_{sample} + m_{water}) \cdot m_{IL}}{\rho_{HPLC} \cdot m_{sample} \cdot (1 - wc_{sample}) \cdot ODW} \qquad \text{(S5)}$$

where C_{HPLC} is the concentration of the analyte calculated using HPLC data in mg·mL^{-1}, m_{sample} is the weight of the IL solution sample added in mg, m_{water} is the weight of the water added to dilute the sample in mg, m_{IL} dry IL weight added during the pretreatment in g, ρ_{HPLC} density of HPLC sample (used value is 1.045 g mL^{-1}), wc_{sample} is water content of the sample.

Table S1: Experimental factors, levels and code of variables chosen for Box-Behnken design

Variable	*Coded Levels*		
	Low level (-1)	Central level (0)	High level (+1)
A: Temperature (° C)	160	170	180
B: Time (minute)	20	30	40
C: IL concentration (wt%)	70	80	90

Table S2: ANOVA results for model fit

Significant model terms	*All terms are significant - Quadratic model*
F - value	74

P - value	<0.0001
Lack of fits	3
R^2	0.986
R^2 *Adjusted*	0.973
R^2 *predicted*	0.965

References

(1) Sluiter, a.; Hames, B.; Ruiz, R.; Scarlata, C.; Sluiter, J.; Templeton, D.; Crocker, D. *Determination of Structural Carbohydrates and Lignin in Biomass*; 2012. https://doi.org/NREL/TP-510-42618.

(2) Shu, Z.; Xie, C.; Zhou, J.; Li, T.; Chen, Y.; Wang, W.; Tan, Y.; Zhao, Z. *Low Solids Enzymatic Saccharification of Lignocellulosic Biomass Laboratory*; 2015; Vol. 747. https://doi.org/10.1016/j.jallcom.2018.03.019.

(3) Luo, X. L.; Zhu, J. Y.; Gleisner, R.; Zhan, H. Y. Effects of Wet-Pressing-Induced Fiber Hornification on Enzymatic Saccharification of Lignocelluloses. *Cellulose* **2011**, *18* (4), 1055–1062. https://doi.org/10.1007/s10570-011-9541-z.

(4) Sluiter, A.; Hames, B.; Hyman, D.; Payne, R.; Scarlata, C.; Sluiter, J.; Templeton, D.; Wolfe, J. *Determination of Total Solids in Biomass and Total Dissolved Solids in Liquid Process Samples*; 2008; Vol. XXV. https://doi.org/NREL/TP-510-42621.
